# Euglycemic SGLT2 Inhibitor–Associated Ketoacidosis in a Nondiabetic Patient: A Case Report

**DOI:** 10.1155/crcc/3970190

**Published:** 2026-10-25

**Authors:** Carmelo Zinnato, Teresa Roatta, Giacomo Berta, Roberto Penso, Vincenzo Russotto

**Affiliations:** ^1^ Department of Anesthesia and Critical Care University Hospital San Luigi Gonzaga University of Turin Turin Italy unito.it; ^2^ Department of Surgical Sciences University of Turin Turin Italy unito.it; ^3^ Department of Oncology University of Turin Turin Italy unito.it

**Keywords:** euglycemic ketoacidosis, ICU, SGLT2 inhibitor–associated ketoacidosis, sodium-glucose cotransporter-2 inhibitors

## Abstract

We present the case of a 62‐year‐old nondiabetic woman receiving dapagliflozin for dilated cardiomyopathy presenting to the emergency department with severe hypoxemic respiratory failure because of suspected cardiogenic pulmonary edema. Despite treatment, she remained symptomatic and required intubation and intensive care admission. After intubation, high‐anion‐gap metabolic acidosis was unmasked. After exclusion of other common causes, SGLT2 inhibitor–associated ketoacidosis due to dapagliflozin was suspected. Marked ketonuria confirmed the diagnosis despite the absence of hyperglycemia. Dapagliflozin was discontinued, and intravenous insulin, glucose infusion, and early enteral nutrition were initiated. Metabolic acidosis resolved completely within 24 h. The patient was successfully extubated and discharged from the intensive care unit 48 h later. SGLT2 inhibitor–associated ketoacidosis should be considered in patients receiving sodium‐glucose cotransporter‐2 inhibitors who develop otherwise unexplained high‐anion‐gap metabolic acidosis, irrespective of diabetic status or blood glucose concentration. Fasting and acute illness are relevant precipitating factors; prompt drug withdrawal, carbohydrate administration, insulin therapy, and nutritional support can lead to rapid resolution.

## 1. Introduction

Sodium‐glucose cotransporter‐2 inhibitors (SGLT2i) increase urinary glucose excretion and were initially approved for the treatment of Type 2 diabetes (T2DM) [1]. Over the past 5 years, their indications have expanded to include patients with heart failure, both with reduced and preserved ejection fraction, irrespective of their diabetes status [2]. Ketoacidosis is an uncommon but potentially life‐threatening adverse effect linked to these drugs (SGLT2i‐associated ketoacidosis). It is mainly driven by glycosuria and a relative insulin‐deficient state, often precipitated by factors such as fasting and acute illness [3]. Albeit rarely, this complication has been recently described also in nondiabetic patients with normal pancreatic function [4]; here, we report another case of SGLT2i‐associated ketoacidosis in a critically ill patient.

## 2. Case Presentation

A 62‐year‐old woman presented to the emergency department with progressive dyspnea and orthopnea since the past 3 days. Her past medical history included multiple sclerosis with flaccid paraplegia, dilated cardiomyopathy with mildly reduced left ventricular ejection fraction (~45%) since March 2024, moderate aortic regurgitation, JAK2‐positive polycythemia, heterozygous MTHFR mutation, depression, and previous breast cancer treated with surgery and radiotherapy. Prior glucose values were within normal limits with no evidence of diabetes. Her chronic medications included pantoprazole, aspirin, pregabalin, duloxetine, carbamazepine, fentanyl (transdermic), sacubitril/valsartan, bisoprolol, dapagliflozin, potassium canrenoate, quetiapine, mirtazapine, and baclofen.

On admission, she was severely hypoxemic (SpO_2_ 67% on room air), mildly hypotensive (80/50 mmHg) without signs of hypoperfusion, and complained of sharp chest pain, worsening with breathing. She denied recent history of fever or cough. Lung ultrasound showed diffuse bilateral B‐lines, whereas chest CT revealed widespread bilateral ground‐glass opacities. Echocardiography demonstrated stable cardiac function compared with previous examinations (left ventricular ejection fraction 45%) without evidence of significant right ventricular dysfunction. Laboratory tests showed mild neutrophilic leukocytosis (WBC 12.500/*μ*L), mildly elevated CRP, and increased NT‐proBNP (5446 pg/mL).

In the first 36 h of hospital stay, she was treated in an intermediate‐care unit, requiring escalating oxygen therapy from Venturi‐mask 60% to high‐flow oxygen therapy, and a trial of helmet CPAP (3 h). Despite an improvement in oxygenation, with an arterial oxygen partial pressure (PaO_2_) to fractional inspired oxygen (FiO_2_) ratio (P/F) of 242 mmHg, she continued to show marked hyperventilation and dyspnea, whereas mild metabolic acidosis was noted on her arterial blood gas (ABG) analysis (see Table 1). The worsening respiratory failure finally required endotracheal intubation and intensive care unit (ICU) admission. Bronchoalveolar lavage and microbiological investigations were negative for infectious pathogens.

**Table 1:** Summary table with timeline main events and ABG values for this patient; pCO_2_ is expressed in mmHg; HCO^3-^ in mmol/L; glycemia in mg/dL.

Time course	Clinical event	Intervention	pH	PCO _ 2 _ (mmHg)	HCO _ 3 _^ − ^ (mEq/L)	BE (mEq/L)	Glycemia (mg/dL)
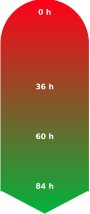	Admission to the ED for hypoxemic respiratory failure	—	7.41	41	26.5	1.8	108
	Admission to ICU for severe respiratory distress requiring intubation	Mechanical ventilation initiated	7.34	37	20.5	−4.8	101
	Worsening metabolic acidosis; urinary ketones positive	Insulin, glucose infusion, and enteral nutrition started	7.30	30	14.9	−10.3	101
	Resolution of metabolic acidosis	Successful extubation	7.43	37	25.5	1.3	129

Following intubation, oxygenation rapidly improved (P/F > 300 mmHg); however, the patient′s metabolic acidosis progressed in the following 24 h (ABG: pH 7.30, pCO_2_ 30 mmHg, pO_2_ 94 mmHg with a FiO_2_ 40%, HCO^3−^ 14.9 mEq/L, BE −10.3). An anion gap of +19 was calculated (Na^+^ 144 mmol/L, Cl^−^ 110 mmol/L, albumin 27 g/L, corrected anion gap ~22). Neither hyperlactatemia nor signs of renal dysfunction were present. Reviewing her medications, ongoing therapy with dapagliflozin was noticed, while she had been fasting for more than 48 h. Urinalysis revealed marked ketonuria (+++), leading to the diagnosis of SGLT2i‐associated ketoacidosis, precipitated by prolonged fasting and critical illness.

Consequently, dapagliflozin therapy was promptly discontinued, and she was treated with an intravenous insulin and glucose infusion (500 mL 5% glucose + 5 U insulin lispro in 4 h). Neither hypoglycemia nor hyperglycemia developed; hypokalemia (due to concurrent furosemide therapy) required multiple corrections, up to 100 mmol in 24 h (lowest value recorded: 3 mmol/L). Enteral nutrition was started at the same time, leading to complete resolution of ketoacidosis within 24 h. The patient also received empirical antibiotics and diuretic therapy for suspected cardiogenic pulmonary edema. She was successfully extubated ~24 h after intubation, bridged with alternating noninvasive ventilation and high‐flow nasal cannula, and discharged from the ICU 48 h later, with the indication to restart dapagliflozin once further improvement of the clinical condition.

## 3. Discussion

The use of SGLT2i is growing and so is the incidence of the adverse effects associated with these drugs [3, 4]. Although first approved as glucose‐lowering drug to treat Type 2 diabetes, their use is increasing among nondiabetic patients, mostly to treat chronic heart failure or chronic renal disease. Ketoacidosis is a well‐known potentially life‐threatening complication of SGLT2i but can be insidious to diagnose. Firstly, despite hyperglycemia being present in more than half cases [5], some patients may have only mildly elevated or even normal glucose levels [6]. Clinical presentation varies widely, ranging from an asymptomatic course to nonspecific symptoms, such as abdominal pain and vomiting, up to severe neurological manifestations, including confusion and dysarthria, or hemodynamic instability requiring vasopressor support [4, 7]. It can be even more challenging if presenting in critically ill, sedated, or comatose patients because of the absence of warning symptoms. Finally, to further complicate the clinical picture, it can also manifest in nondiabetic subjects.

The first two cases of SGLT2i‐associated ketoacidosis in nondiabetic patients were described by Umapathysivam et al. in 2023 [8]. In both cases, pH and serum bicarbonate levels were lower than those observed in our patient. Reduced oral intake due to vomiting and diarrhea was identified as the precipitating factor. Although our patient did not present with such symptoms, reduced oral intake secondary to concurrent respiratory failure was considered the main precipitating factor. This view is supported by previous data [9] which identifies the time of the last meal as the strongest predictor of ketonemia. Furthermore, SGLT2i therapy was not withheld until the diagnosis was established—more than 48 h after presentation to the emergency department. Despite dapagliflozin being well tolerated since its start around 2 years before the current presentation, this case shows the risk of continuing these medications in such situations. To our knowledge, only few other cases of SGLT2i‐associated ketoacidosis in nondiabetic patients have been described so far [4, 7]; most of them included fasting (as before surgery) or a reduced oral intake, combined with different “stressors,” as potential triggering factors. Our case is, in our view, unique as it shows the role of ketoacidosis and subsequent hyperventilation as a contributing factor for respiratory decompensation requiring endotracheal intubation, in a patient with an underlying respiratory failure due to cardiac causes.

The mechanisms through which SGLT2i cause ketoacidosis are thought to be related to persistent drug‐induced glycosuria, which causes metabolic shift to enhanced usage of fat for energy production (see Figure 1). This is because of changes in hormonal homeostasis with an increased glucagon‐to‐insulin ratio, leading to a number of metabolic consequences including increased lipolysis, decreased glucose utilization, increased fatty acid oxidation, and ketogenesis [10]. These changes have been described both in T2DM patients and in nondiabetic patients, though in the latter to a lesser extent and with attenuated mechanism. A second mechanism that explains the metabolic acidosis in patients treated with SGLT2i is the altered ATP turnover at renal level, as the filtered ketoacids are eliminated as salts of Na+ or K+, subsequently leading to indirect bicarbonate loss [10]. Risk factors for developing SGLT2i‐associated ketoacidosis are a sudden reduction in exogenous insulin dose or inadequate endogenous insulin secretion, fasting, metabolic stress, female sex, dehydration, surgery, and infections [11].

**Figure 1:**
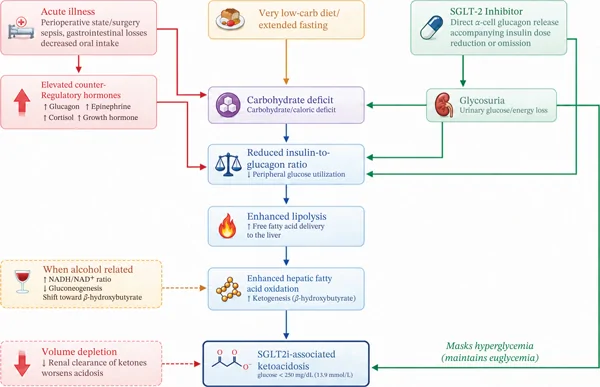
Mechanisms of SGLT2i‐associated ketoacidosis development in critically ill patients.

Upon admission in the ED, our patient showed on ABG analysis normal pH and bicarbonate levels, whereas the main concern was the severe hypoxemia. She developed a progressively worsening metabolic acidosis with high anion gap starting from approximately 36 h from admission. According to the Stewart approach to acid‐base physiology, in the presence of normal PaCO_2_, normal sodium and chloride levels and the metabolic acidosis could be explained by the accumulation of unmeasured anions, such as ketone bodies. For this reason, a urinalysis was performed despite normal glucose levels, revealing elevated levels of ketone bodies in the urine, confirming the suspicion of SGLT2i‐associated ketoacidosis. However, urine dipstick testing predominantly detects acetoacetate rather than *β*‐hydroxybutyrate, the principal circulating ketone body and a more reliable marker of ketonemia [12]. One limitation of this case report is the unavailability of *β*‐hydroxybutyrate measurement, which is considered the reference method. Urinary ketones measurement presents some limitations; in fact, the kinetics of acetoacetate elimination does not follow plasmatic levels of *β*‐hydroxybutyrate, lagging behind serum values. It can therefore be falsely negative at the beginning and persistently positive despite adequate treatment. In addition, SGLT2i can increase ketone reabsorption, reducing the diagnostic values of urinary dipstick.

Treatment of diabetic ketoacidosis finds its natural treatment in insulin administration, whose absence is responsible for the metabolic derangements. In nondiabetic patients with normal pancreatic function, insulin secretion is thought to be adequate if adequately stimulated, such as after meal consumption. Nonetheless, our patient was treated with iv insulin and glucose; at the same time, enteral nutrition was started via a nasogastric tube. We believe this was the cornerstone of treatment. In fact, this led to a rapid and complete resolution of the metabolic acidosis within 24 h.

## 4. Conclusion

Ketoacidosis is a well‐known complication of SGLT2i, but its presentation can be subtle, especially in the absence of overt hyperglycemia. The use of these medications in nondiabetic patients is growing because of indications such as chronic heart failure; despite normal pancreatic function, these patients are at risk of ketoacidosis due to a drug‐induced shift toward ketogenesis. High‐anion‐gap metabolic acidosis is the hallmark of SGLT2i‐associated ketoacidosis, and its presence should always warn the clinician of this possible differential diagnosis.

## Author Contributions

C.Z., T.R., and V.R. treated the patient and conceived the case description; C.Z., T.R., G.B., R.P., and V.R. provided important intellectual contents and contributed to manuscript preparation.

## Funding

No funding was received for this manuscript.

## Disclosure

All authors approved the final version of the manuscript.

## Ethics Statement

An ethics committee approval was not required for this case report description. Patient′s informed consent for publication was obtained.

## Conflicts of Interest

The authors declare no conflicts of interest.

## Supporting information


**Supporting Information** Additional supporting information can be found online in the Supporting Information section.. File S1: CARE checklist of information to include when writing a case report.

## Data Availability

Data sharing is not applicable to this article as no datasets were generated or analyzed during the current study.
